# Supplementary material for: Metamaterial-enabled asymmetric negative refraction of GHz mechanical waves
Source: Nat Commun. 2022 Oct 8;13:5939. doi: 10.1038/s41467-022-33652-8 (PMC9547911; doi:10.1038/s41467-022-33652-8)
Supplement: Supplementary file 1 — Supplementary Information [file 41467_2022_33652_MOESM1_ESM.pdf]

Supplementary Information for the article

"Metamaterial-enabled asymmetric negative refraction of GHz mechanical waves"

by S. Zanotto, G. Biasiol, Paulo V. Santos, A. Pitanti

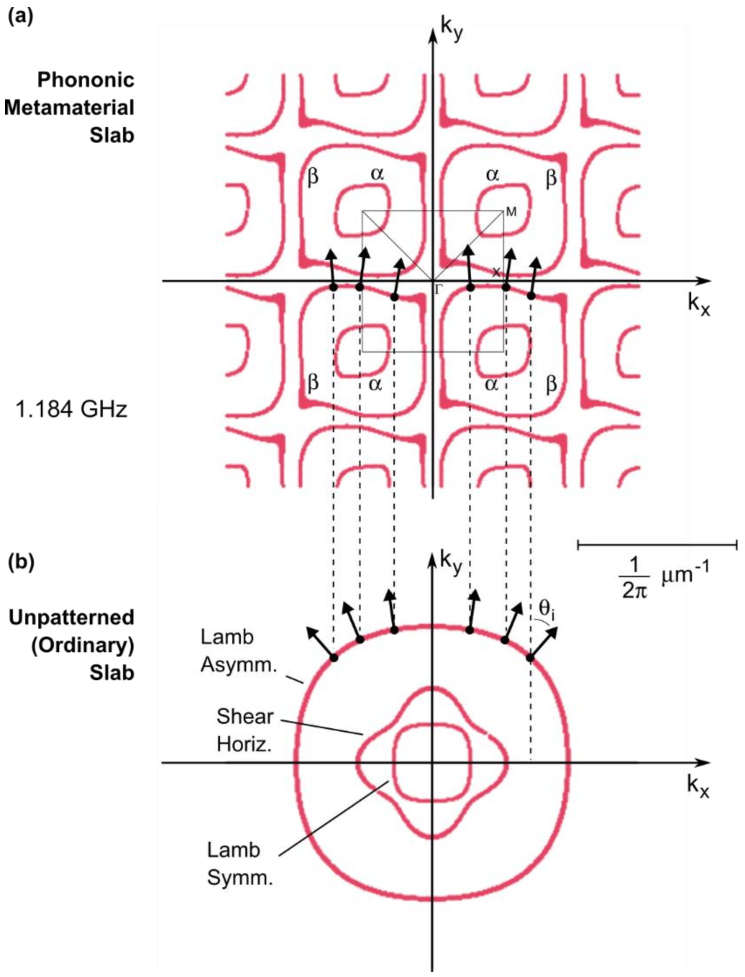

Supplementary Figure 1. Quantitative representation of isofrequency curves. In Fig. 1c-d of the main text we reported a conceptual diagram representing the asymmetric negative refraction process. As the aim of the aforementioned figure is to highlight specific aspects of the problem, it is lacking a quantitative simultaneous representation of unpatterned slab and metamaterial slab isofrequency curves. We report such a quantitative representation in Suppl. Fig. 1 (a-b). Panel (b) also provides the link between the k_x wavevector component and the wave incident angle; it is upon this link that the double horizontal axis of Fig. 4a has been built. The metamaterial isofrequency curves have been plotted in the extended Brillouin zone scheme, truncated at wavevector values that are sufficiently large not to be involved in the refraction process.

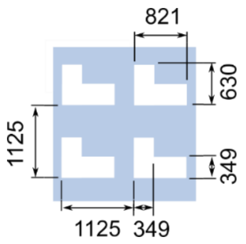

Supplementary Figure 2. Geometrical parameters defining the L-shaped holes that constitute the mechanical metamaterial. Here we report the relevant measures, in nanometers, defining the metamaterial L-shaped holes. They are drilled in a 210 nm thick gallium arsenide membrane.

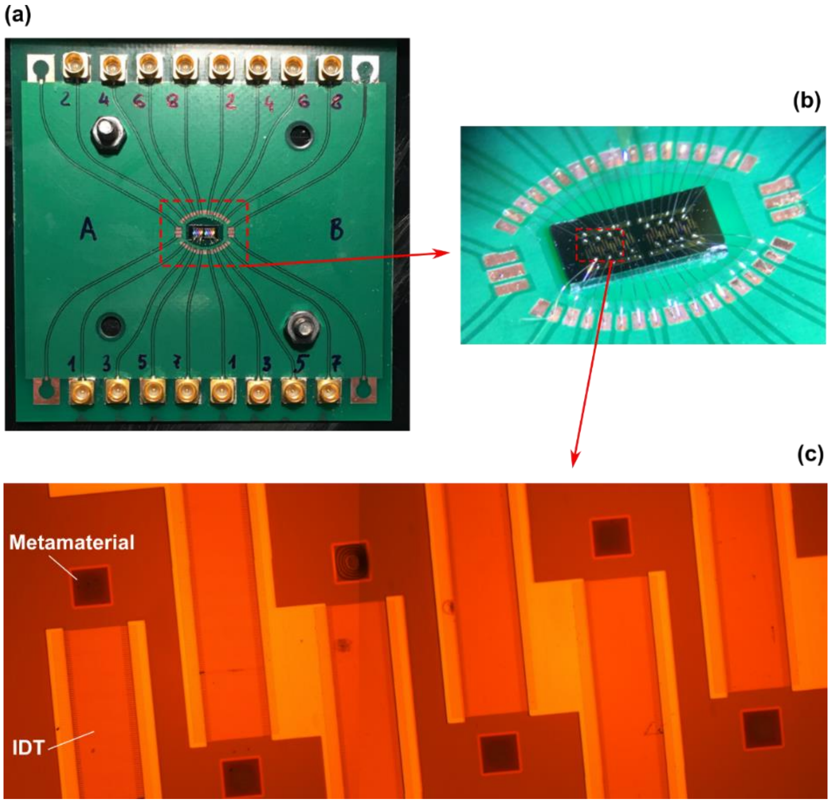

Supplementary Figure 3. Details of sample and sample mounting. Here we report an overall view of the PCB carrying the sample (a); a detail of the sample and of bonding wires (b); and a microscope view of an array of different-pitch IDTs, each facing an identical metamaterial (c).

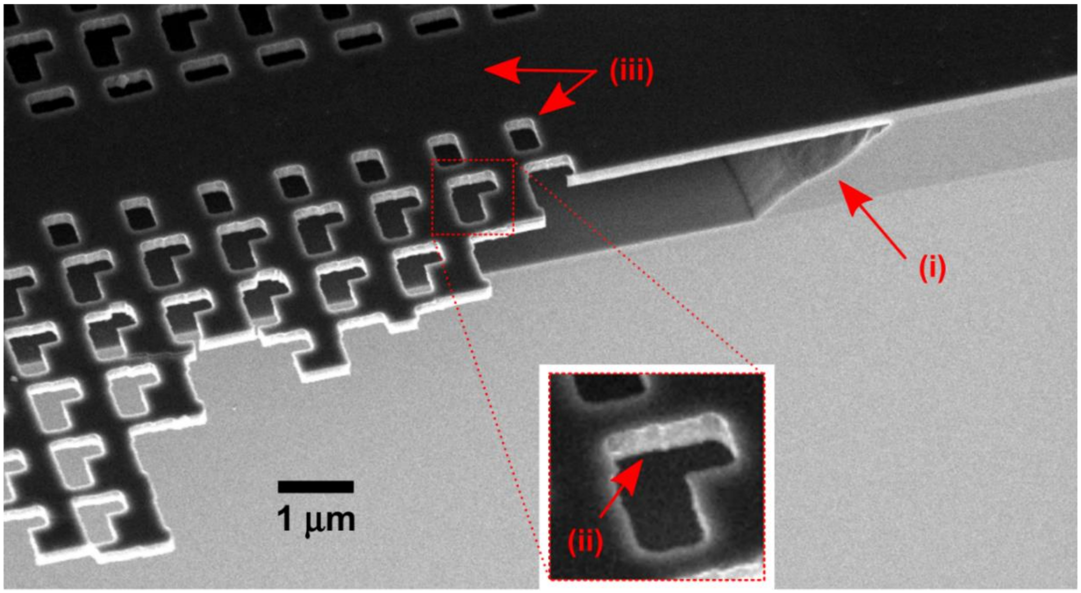

Supplementary Figure 4. Analysis of the imperfections leading to wave scattering at the metamaterial interface. We report here a scannning electron micrography of the cross-section of a sacrificial sample, fabricated with the same technique as the samples analyzed in the main text (Figs. 2 and 3). In (i) it is visible that the interface between the underetched air spacer and the bulk supporting chip is affected by some roughness (originating from the wet etching process step). In (ii) some irregularities on the vertical hole sidewalls are visible, as well as some deviations of the L-hole shape from the ideal polygon (these effects originate from the electron-beam lithography and the dry etching process steps). The missing hole line and wrong hole shape in (iii) originate from lithography errors; these kind of imperfections were not present in the samples analyzed in the main text.

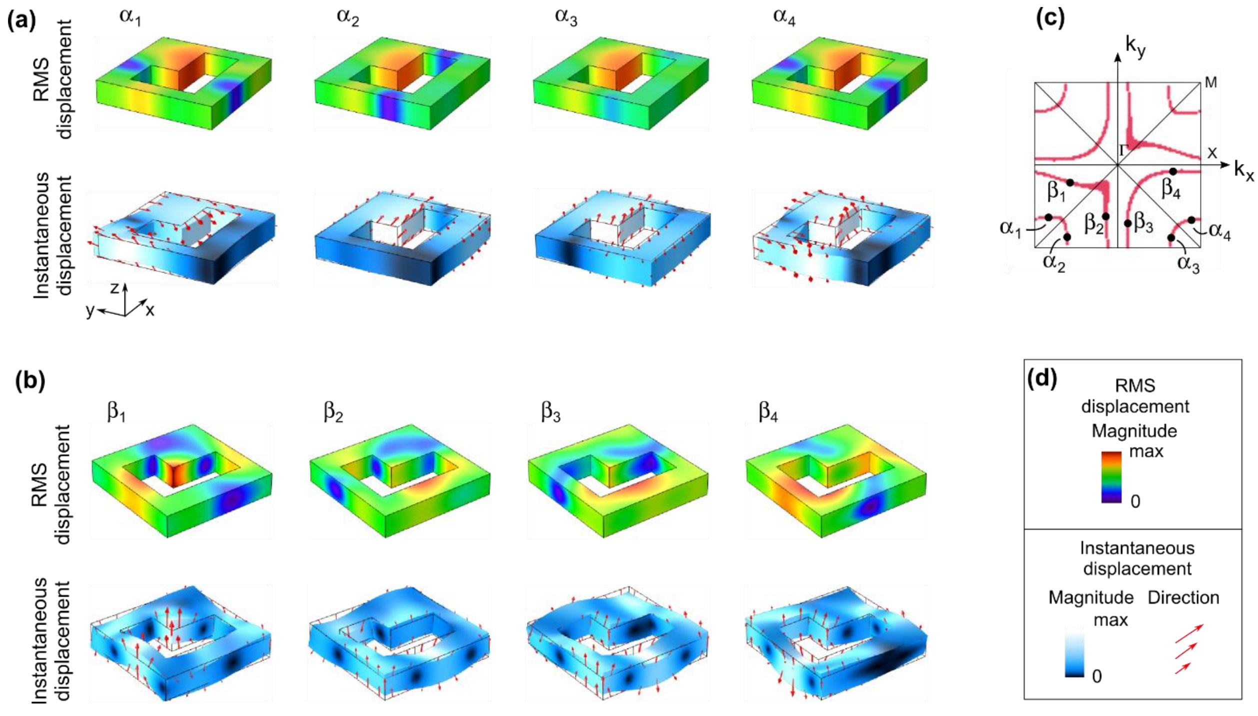

Supplementary Figure 5. Displacement maps for the relevant mechanical modes. In panels (a) and (b) we plot displacement maps calculated for several points in the first Brillouin zone, chosen from the isofrequency curves labeled as α and β in Figs. 3c and 3f of the main text, see also panel (c) here. From (a) it clearly appears that the displacement for modes α is mostly in-plane (i.e., it has almost only *x*- and *y*- components), while from (b) it results that for modes β the strongest component is that along *z*. In other words, mode α is shear-horizontal-like, while mode β is Lamb-asymmetric-like. We attribute to this difference between modes α and β the discrepancy observed between experimental and computational data in Figs. 3c and 3f of the main text, i.e., the fact that mode α is not visible in the experiment. Indeed, mode α is hardly excited by the SAW driving wave. The data in the Suppl. Fig. 5 have been calculated at a frequency of 1.184 GHz, thus corresponding to main Figs. 3c and 3f; however, the data shown here also support the mismatch observed between main Figs. 3b and 3e (1.024 GHz). In those Figures, the feature indicated by the arrow, that is only faintly visible in the experiment, originates from an isofrequency curve that is the continuation of mode α, and that shares with it the prevalent in-plane motion character. In Suppl. Fig. 5 the instantaneous displacement has been calculated in correspondence to the time that maximizes the displacement within the acoustic wave period (see (d) for colormap legend).
